# B cells at the adaptive-innate immune system interface in SLE

**DOI:** 10.1186/ar3958

**Published:** 2012-09-27

**Authors:** JH Anolik, A Palanichamy, J Bauer, J Barnard, J Biear, R Dedrick, I Sanz, J Liesveld, E Baechler

**Affiliations:** 1 University of Rochester Medical Center, Rochester, NY, USA; 2 University of Minnesota, Minneapolis, MN, USA; 3 XDx, Brisbane, CA, USA; 4 Emory University, Atlanta, GA, USA

## Background

Accumulating data indicate that inappropriate activation of type I interferon (IFN) plays a key role in the pathogenesis of systemic lupus erythematosus (SLE). Given that IFN can influence B-cell lymphopoiesis in murine bone marrow (BM), we explored the hypothesis that IFN activation in SLE BM has direct effects on B-cell development. Additionally, the impact of B cells on pDC production of IFN was examined.

## Methods

Peripheral blood mononuclear cells (PBMC) and bone marrow mononuclear cells (BMMC) were isolated from 28 patients fulfilling ACR criteria for SLE and from 20 healthy controls. RNA isolates were analyzed for the expression of three to five IFN-regulated genes (IFIT1, IRF7, G1P2, CEB1, C1orf29) by quantitative PCR and IFN-regulated chemokines CXCL10 (IP-10), CCL2 (MCP-1), and CCL19 (MIP-3β) defined in serum and BM supernatant. B-cell subsets were delineated in single-cell suspensions of PBMC and BMMC by multi-parameter flow cytometry. BM and PB pDCs were stimulated with TLR9 (CpG ODN 2006 or 2216) or TLR7 (R848) agonists and intracellular IFN and TNF measured by flow cytometry.

## Results

The majority of SLE patients had an IFN signature in the BM (57%), which was even more pronounced than the paired PB. Notably, the early B-cell compartment (consisting of pro B cells, pre B cells, immature B cells and early transitional T1 and T2 B cells) in SLE BM with an IFN signature was associated with a reduction in the fraction of pro/pre B cells, suggesting an inhibition in early B-cell development. However, at the transitional B-cell stage this inhibition was reversed with enhanced selection of B cells into the transitional compartment. The composition of the mature B-cell compartment in IFN-activated SLE BM was notable for an expansion of CD27^+^, IgD^- ^switch memory B cells. SLE patients with a BM IFN signature were enriched for a high number of autoantibody specificities (*P *= 0.003 compared with IFN low), and the degree of IFN activation in the BM correlated with peripheral lymphopenia (*P *= 0.019) and disease activity (*P *= 0.05). In order to understand the etiology of IFN activation in SLE BM, we examined the production of IFN by pDCs. CpG induced IFN production in pDCs in a dose-dependent fashion. Notably, a higher proportion of BM pDCs produced IFN compared with paired PB. Moreover, pDCs produced 59% more IFN in the presence of B cells.

## Conclusion

This is the first demonstration of an IFN signature in SLE BM. These results suggest that the BM is an important but previously unrecognized target organ in SLE with critical implications for B-cell ontogeny and selection. We postulate that in the setting of IFN activation the stringency of negative selection of autoreactive B cells in the BM may be reduced. Circulating immune complexes and apoptotic fragments in SLE BM may serve as ligands for Toll-like receptors on pDCs contributing to aberrant IFN production and, in turn, B cells may be critical regulators of pDC function.

